# The complete chloroplast genome of *Euonymus alatus* (Celastraceae)

**DOI:** 10.1080/23802359.2022.2067494

**Published:** 2022-04-25

**Authors:** Kun Ning, Ting Zhou, Yaolong Wang, Yousry A. El-Kassaby, Yan Ma

**Affiliations:** a College of Horticulture, Jinling Institute of Technology, Nanjing City, P. R. China; b Institute of Botany, Jiangsu Province and Chinese Academy of Sciences (Nanjing Botanical Garden Mem. Sun Yat-Sen), Nanjing City, P. R. China; c Department of Forest and Conservation Sciences, Faculty of Forestry, University of British Columbia, Vancouver, Canada

**Keywords:** *Euonymus alatus*, chloroplast genome, phylogeny

## Abstract

*Euonymus alatus*, Celastraceae, is a deciduous tree species valued for its ornamental and medicinal properties. Here, the species’ whole chloroplast genome sequence was generated by assembling the Illumina paired-end sequencing reads. The circular genome was 157,611 bp in length, exhibiting a typical quadripartite structure with a large single-copy (LSC: 85,892 bp), a small single-copy (SSC: 18,419 bp), and a pair of inverted repeat regions (IRA and IRB: each of 26,650 bp). The chloroplast genome encoded 131 genes, including 87 protein-coding (78 protein-coding gene species), 36 transfer RNA (29 tRNA species), and 8 ribosomal RNA genes (4 rRNA species). The overall GC content was 37.3%, while the corresponding values of the LSC, SSC and IR regions were 35.1, 31.7 and 42.7%, respectively. Phylogenetic analysis of 12 species complete chloroplast genomes suggested that *E. alatus* was relatively close to *E. japonicus*. This complete chloroplast genome is expected to provide valuable insight into further phylogenetic reconstruction of the Celastraceae species.

## Introduction

*Euonymus alatus* is a deciduous tree species in the Celastraceae family, distributed mainly in China and East Asia (Qin et al. 2011). The species is not only precious for its high ornamental attributes in landscaping and valued for its medicinal properties, such as wide pharmacological effects of anti-diabetes, anti-inflammatory and tumor (Fan et al. 2020). Generating genetic information of *E. alatus* is expected to facilitate better understanding of ecological and pharmacological studies. Here, we assembled the complete chloroplast genome sequence of *E. alatus*, aiming at expanding the genetic evolutionary histories for the *Euonymus* species.

The *E. alatus* specimen was deposited at the herbarium of Jinling Institute of Technology (Jiangsu Province, China; 32°13’E, 118°82’N; Accession number: JKY02312). For total genomic DNA extraction, fresh leaves were collected from an *E. alatus* individual and Hi-DNAsecure Plant Kit (Tiangen Biotech Co., Ltd, Beijing, China) was utilized. After that, paired-end reads of 150 bp were sequenced through the Illumina HiSeq 4000 platform. Genome assembly was performed by SPAdes v3.13.0 (Bankevich et al. 2012) with *E. japonicus* (GenBank accession number NC028067) as the reference. The chloroplast genome was annotated using GeSeq and CPGAVAS2 software (Tillich et al. 2017; Shi et al. 2019).

The complete chloroplast genome of *E. alatus* (GenBank accession number OK562424) was 157,611 bp in length, with a large single-copy region (LSC) of 85,892 bp, a small single-copy region (SSC) of 18,419 bp, and two inverted repeat (IR) regions of 26,650 bp. The circular chloroplast genome contained 131 genes, including 87 protein-coding (78 protein-coding gene species), 36 tRNA (29 tRNA species) and 8 rRNA genes (4 rRNA species). The overall GC content was 37.3% and the corresponding values of LSC, SSC, and IR regions were 35.1%, 31.7%, and 42.7%, respectively.

**Figure 1.**
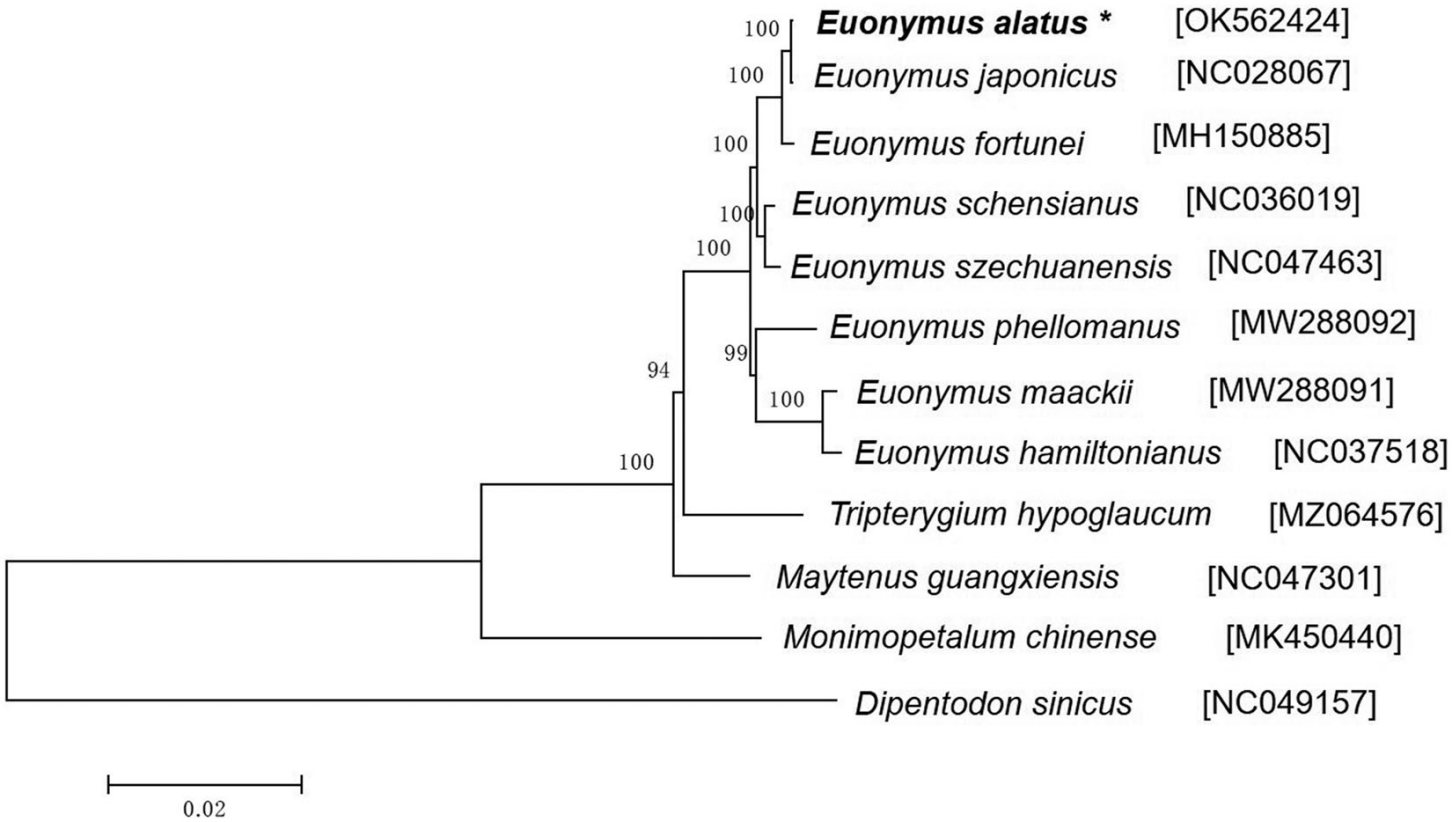
Phylogenetic tree constructed by maximum-likelihood (ML) method based on complete chloroplast genome sequences from *E. alatus* and other species. *Dipentodon sinicus* was selected as an outgroup, and analyses with 1000 bootstrap replicates were used to calculate the bootstrap values.

Phylogenetic trees are widely used in genetic and evolutionary research (Zhang et al. 2021) and we utilized the complete chloroplast genomes of 12 species to construct a phylogenetic tree using the maximum-likelihood (ML) method based on GTR + I model in MEGA v7.0.14 with 1000 bootstrap replications (Kumar et al. 2016). All sequences were aligned by the MAFFT (Katoh and Standley 2013). The phylogenetic analysis indicated that *E. alatus* was closely related to *E. japonicus* when compared with other Celastraceae species (Figure 1). We believe that the generated sequence will provide valuable insight into the classification and evolutionary histories for *E. alatus* species.

Altogether, the value insights we gained in this study: (1) Details of the chloroplast genome in *E. alatus.* (2) *E. alatus* was relatively close to *E. japonicus* among the Celastraceae species we analyzed. (3) *E. alatus*’ chloroplast genome will contribute to further phylogenetic reconstruction of the Celastraceae species.

## Statements

1. This study did not involve any ethical issues, so no ethics committee or relevant permissions were required.
2. The name of species in full and the authority (__Euonymus alatus__ Thunberg 1830).
3. The *Euonymus alatus* specimen was deposited at the herbarium of Jinling Institute of Technology (https://yyx.jit.edu.cn/index.htm Contact person: Yan Ma Email: JITmayan@163.com) under the voucher number JKY02312.

## Data Availability

The genome sequence data that support the findings of this study are openly available in GenBank of NCBI at (https://www.ncbi.nlm.nih.gov/) under the accession no. OK562424. The associated BioProject, SRA, and Bio-Sample numbers are PRJNA785676, SRR17106723, and SAMN23574701 respectively.
